# Graphene-TLL-Cu_2_ONPs Hybrid as Highly Efficient Catalyst for Degradation of Organic Compounds

**DOI:** 10.3390/nano13030449

**Published:** 2023-01-21

**Authors:** Noelia Losada-Garcia, Jannier Carranza, Jose M. Palomo

**Affiliations:** Instituto de Catálisis y Petroleoquímica (ICP), CSIC, Marie Curie 2, 28049 Madrid, Spain

**Keywords:** graphene, copper oxide nanoparticles, nanohybrid, trichloroethylene, Rhodamine B, water remediation

## Abstract

In this work, Cu_2_O nanoparticles (NPs) were created in situ on graphene functionalized with *Thermomyces lanuginosus* lipase (G@TLL) where site-oriented supported TLL acted as template and binder in the presence of copper salt by tailorable synthesis under mild conditions, producing a heterogeneous catalyst. Cu_2_O NPs were confirmed by XRD and XPS. The TEM microscopy showed that the nanoparticles were homogeneously distributed over the G@TLL surface with sizes of 53 nm and 165 nm. This **G@TLL-Cu_2_O** hybrid was successfully used in the degradation of toxic organic compounds such as trichloroethylene (TCE) and Rhodamine B (RhB). In the case of TCE, the hybrid presented a high catalytic capacity, degrading 60 ppm of product in 60 min in aqueous solution and room temperature without the formation of other toxic subproducts. In addition, a TOF value of 7.5 times higher than the unsupported counterpart (TLL-Cu_2_O) was obtained, demonstrating the improved catalytic efficiency of the system in the solid phase. The hybrid also presented an excellent catalytic performance for the degradation of Rhodamine B (RhB) obtaining a complete degradation (48 ppm) in 50 min in aqueous solution and room temperature and with the presence of a green oxidant as H_2_O_2_.

## 1. Introduction

Water is one of the valuable resources necessary to sustain life on Earth. It covers about 70.9% of the total surface of the Earth. However, 3% of all water on Earth is present as fresh water. This small amount of the total water is used for various purposes, e.g., drinking, agricultural and domestic use. However, many industries directly discharge their wastes (toxic and harmful substances) into fresh water [1,2,3]. These substances are constantly circulating in the environment and continue to endanger the earth through different pathways [4]. Among them, industrial wastewater is particularly harmful to the environment, so it makes sense to find effective ways to treat the organic pollutants present since most of them come from the textile, plastic, paint and pharmaceutical industries, and they are classified as poorly biodegradable and highly toxic [5,6,7]. Specifically, trichlorethylene (TCE) is one of the predominant chlorinated organic compounds that has been widely used in commercial and industrial fields [8,9]. However, it has generated widespread contamination of groundwater and soil, which is why TCE is classified as a Group 1 human carcinogen by the International Agency for Research on Cancer (IARC) [10]. According to the United States Environmental Protection Agency (EPA), the control standard for TCE is 0.0050 mg/L [11].

On the other hand, Rhodamine B (RhB) is a widely used organic compound defined as a cationic basic dye, which belongs to anthraquinone [12]. Wastewater produced by this dye is characterized by high chromaticity, difficult biochemical degradation, and a high concentration of organic pollutants [13].

To address this problem, in recent decades, researchers have used a number of chemical and physical processes including coagulation, flocculation, membrane filtration, and adsorption, all of which are inefficient and expensive, generating by-products that require post processing [14,15,16,17]. Therefore, the use of metallic nanoparticles has been one of the new methodologies used in the degradation of organic pollutants in water. Among the various transition metals under study, copper nanoparticles (CuNPs) are less expensive compared to noble metals such as platinum, silver and gold, in addition to presenting important physical and chemical properties, catalytic properties, such as photocatalysis, optical and magnetic, and heat transfer, high surface area/volume ratio, antibacterial potency, and biocidal properties [18,19]. Therefore, CuNPs could be used as effective candidates for the removal of polluting organic compounds from the environment. However, the synthesis of CuNPs in many cases suffers from aggregation and difficult recovery due to their small size and high surface energy [20,21]. In order to overcome these problems and therefore be able to stabilize these nanoparticles, different alternatives have been developed in recent years, such as the direct incorporation of metals, such as supports or organometallic complexes in the structure of a protein (creating new artificial active sites) [22,23,24,25,26], and the direct in situ formation of metal nanoparticles induced by the enzymatic structure to create new nanozymes, a pioneering idea by our group [27,28,29].

This last strategy has been demonstrated with different enzymes, especially with lipases. By following this strategy, it is possible to obtain directly heterogeneous catalysts, because of the direct enzyme aggregation during the metal nanoparticles synthesis. This phenomenon could block some metallic catalytic active sites, reducing their efficiency in several processes.

In our group, we recently developed a strategy based on the preparation of an enzyme derivative, where the enzyme is previously selectively adsorbed on graphene sheets [30]. This support allows us to fix the enzyme exclusively in open conformation [31], obtaining all enzyme molecules homogenously distributed on the solid phase, thus avoiding protein aggregation. This could allow us to achieve one particle of metal per protein molecule, thus making all metallic active sites accessible. Additionally, graphene offers a large specific surface area and high electronic conductivity, biocompatibility, and chemical stability [32], in addition to the possibility of interactions with some organic molecules facilitating the catalytic performance.

In this work, a solid-phase nanohybrid formed by in situ synthesized copper oxide nanoparticles induced by *Thermomyces lanuginosus* lipase (TLL) previously immobilized on multi-layers’ graphene support was synthesized and applied for the degradation of toxic organic compounds in water (TCE and RhB) under sustainable conditions.

## 2. Materials and Methods

### 2.1. Chemicals 

*Thermomyces lanuginosus* solution (Lipozyme^®^ TL 100L) was from Novozymes (Copenhagen, Denmark). Copper (II) sulfate pentahydrate, sodium acetate and hydrogen peroxide (33%, H_2_O_2_) were from Panreac (Barcelona, Spain). Graphite flakes, sodium bicarbonate, sodium phosphate, sodium borohydride, Rhodamine B (RhB), trichloroethylene (TCE), 1,1-dichloroethylene (1,1-DCE), and vinyl chloride (VC) were purchased from Merck (Darmstadt, Germany). HPLC grade acetonitrile, methanol, tetrahydrofurane (THF) and dioxane (98%) were from Scharlau (Barcelona, Spain). Tween-80, dodecyl sulphate sodium (85%), Mercaptoethanol and cetyl trimethyl ammonium bromide (CTAB) were purchased from Thermo Fischer Scientific (Waltham, MA, USA).

### 2.2. Structural Characterization

The metal contents were measured by an inductively coupled plasma–optical emission spectrometer (ICP-OES) (OPTIMA 2100 DV instrument; PerkinElmer, Waltham, MA, USA). X-Ray diffraction (XRD) patterns with Cu Kα radiation (Texture Analysis D8 Advance Diffractometer; Bruker, Billerica, MA, USA) were used for structure characterization. Transmission electron microscopy (TEM) and high-resolution TEM microscopy (HR-TEM) images (2100F microscope; JEOL, Tokyo, Japan) were used to measure the size and distribution of the samples. Interplanar spacing in the nanostructures was calculated by using the inversed Fourier transform with the GATAN digital micrograph program (Corporate Headquarters, Pleasanton, CA, USA). X-ray photoelectron analysis (XPS) was carried out on SPECS GmbH spectrometer equipped with Phoibos 150 9MCD energy analyser. A nonmonochromatic magnesium X-ray source with a power of 200 W and voltage of 12 kV was used. FTIR spectra were recorded on FT-IR 470-Series (JASCO) spectrophotometer using glass of germanium. To recover the bionanohybrid, a Biocen 22 R (Orto-Alresa, Ajalvir, Spain) refrigerated centrifuge was used. Spectrophotometric analyses for RhB reactions were run on a V-730 spectrophotometer (JASCO, Tokyo, Japan). A HPLC pump PU-4180 (JASCO, Tokyo, Japan) was used to analyse the TCE reactions. Analyses were run, at 25 °C, using a UV-4075 UV/Vis detector (JASCO, Tokyo, Japan). 

### 2.3. Synthesis of G@TLL-Cu_2_O Hybrid

An amount of 500 mg of G@TLL (containing 3 mg of TLL) was added to 8 mL buffer 0.1 M of sodium phosphate pH 7) in a 75 mL glass vial containing a small magnetic bar stirrer. Then, 80 mg of Cu_2_SO_4_ · 5H_2_O (10 mg/mL) was added to the solution with the support, and it was maintained for 16 hours. After 16 h, 800 µL of NaBH_4_ (45 mg) aqueous solution (1.2 M) was added to the black solution obtaining a final concentration of 0.12 M sodium borohydride in the mixture. The mixture was reduced for 30 min. After incubation, the mixture was centrifuged at 8000 rpm for 20 min. The sediment generated was re-suspended in 5 mL of water. It was centrifuged again at 8000 rpm for 20 min, and the supernatant was removed. The process was repeated two more times. Finally, the supernatant was removed, and the pellet was re-suspended in 2 mL of water and added to a cryogenisation tube, frozen with liquid nitrogen and lyophilized overnight. After that, the so-called **G@TLL-Cu_2_O** was obtained.

### 2.4. Experiments of G@TLL-Cu_2_O Hybrid Enzyme Desorption on Graphene Support

An amount of 20 mg of **G@TLL-Cu_2_O** was incubated in 1 mL of aqueous solution containing for CTAB (1% *v*/*v*), acetonitrile (10, 50, 70 or 90% *v*/*v*), Tween 80 (2% *v*/*v*), and Triton-X100 (2% and 5% *v*/*v*) for 1 h. Then, supernatant and suspension was analysed by enzymatic activity assay. The solid was filtered and analysed by XRD. Two additional experiments were performed, at 90 °C and 50 °C, for 1 h with water, and other with electrophoretic native degradation buffer (SDS). The solids were also analysed by XRD. 

### 2.5. G@TLL-Cu_2_O Hybrid Catalysing the Degradation of Trichloroethylene (TCE)

A solution of 10 mM of TCE in pure acetonitrile was prepared. Then, 0.2 mL of this solution was dissolved in 2 mL of 50:50 pure ACN: distilled water, 100 mM of buffer sodium acetate pH4, buffer sodium phosphate pH7 or buffer sodium bicarbonate pH 8.5 to achieve a concentration of 1 mM TCE (131.4 mg/L). Then, in some cases, hydrogen peroxide (33% *v*/*v*) was added to this TCE solution to obtain a concentration of 10eq. To initialize the reaction, 1–10 mg of the catalyst was added to this solution (TCE or TCE + H_2_O_2_) in a 7 mL glass flask. Gentle stirring was provided at room temperature (r.t., 25 °C) and natural light by a roller. At given time intervals, 20 µL of reaction solution was taken and diluted in 180 µL of acetonitrile pure for HPLC analysis. For each degradation, three parallel experiments were performed, and the error range was <5%. HPLC conditions were 50:50 acetonitrile: milliQ water at 1 mL/min, λ:215 nm using UV-Vis detector. Under these conditions, the retention time of TCE was 9 min. TCE subproducts such as 1,1-DCE or vinyl chloride were detected at 7 min and 4 min, respectively, using commercial standard pure products of both substrates as control. 

TOF (turnover frequency) value was calculated by [mmols TCE converted/mmols Cu × time (min)^−1^].

### 2.6. G@TLL-Cu_2_O Hybrid Catalysing the Degradation of Rhodamine B (RhB)

A solution of 0.1 mM (48 mg/L) of RhB in 50:50 pure ACN: distilled water, 100 mM of buffer sodium acetate pH4, buffer sodium phosphate pH 7 or buffer sodium bicarbonate pH 8.5 was prepared. Then, 2 mL of this solution was mixed with 0–250 mM of H_2_O_2_. To initialize the reaction, 5–10 mg of catalysts was added to this solution (RhB or RhB + H_2_O_2_) in a 7 mL glass flask. Gentle stirring was provided at room temperature (r.t., 25 °C) and natural light by a roller. At given time intervals, 50 µL of reaction solution was taken and diluted in 1950 µL of distilled water measuring the absorption spectrum between 800 and 300 nm in a spectrophotometer with maximum absorbance at 550 nm.

## 3. Results and Discussion

### 3.1. Preparation and Characterization of G@TLL-Cu_2_O Hybrid

Graphite flakes were initially used to obtain graphene with immobilized lipase (G@TLL) [30]. This strategy ensures the presence of lipase by fixing the open conformation and homogeneously dispersed on the solid material. This is due to the site-oriented interactions between hydrophobic graphene surface with particularly hydrophobic pocket area in the open conformation of lipase constitute by surrounding active site and lid area (Figure S1A). *Thermomyces lanuginosus* lipase (TLL), a typical lipase which mainly exists as dimeric form in solution was used. The more hydrophobic area around the active site and a larger oligopeptide lid of this enzyme allowed us to create an immobilized G@TLL with better graphene exfoliation. Complete immobilization was achieved after 40 min incubation at room temperature (Figure S1B). The enzyme adsorbed on the graphene support showed even higher activity compared to soluble one (Figure S1C), due to fixing open monomeric conformation of the enzyme on the solid phase compared to the dimeric soluble form.

**Figure 1:**
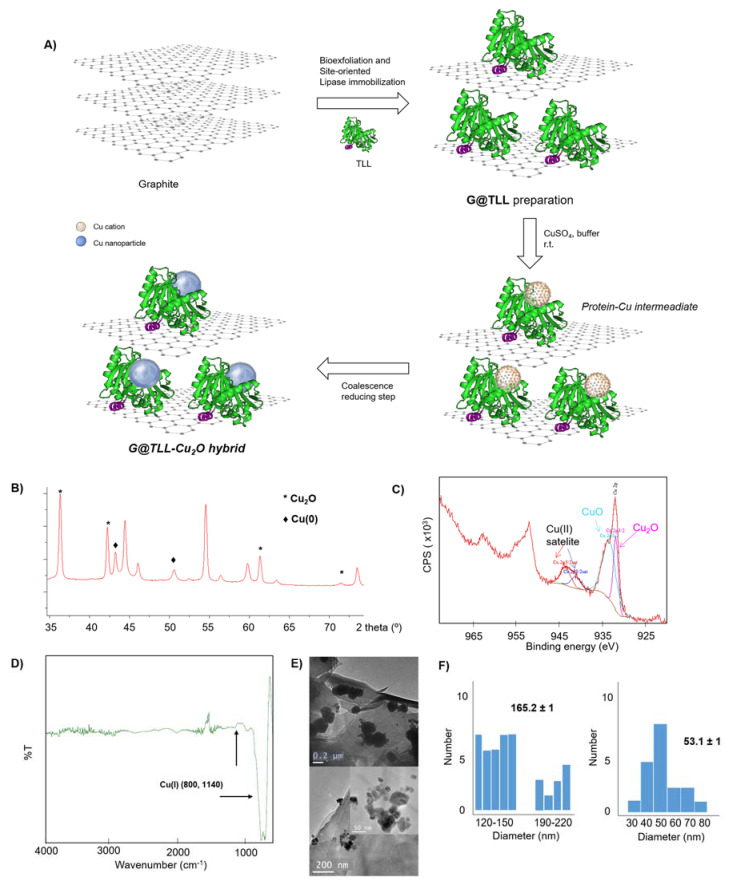
(**A**) **G@TLL-Cu_2_O** hybrid preparation scheme; (**B**) XRD spectrum; (**C**) XPS spectrum of Cu2p; (**D**) FT-IR spectrum; (**E**) TEM images and HR-TEM image (inset); (**F**) Nanoparticles size distribution.

The synthesis of **G@TLL-Cu_2_O** heterogeneous hybrid was performed as shown in Figure 1A. Copper nanoparticles (CuNPs) were synthesized by a mild-aqueous methodology, using copper sulphate and where site-oriented supported TLL acted as template and binder. The preparation started by mixing G@TLL in a phosphate solution at pH 7 with the copper salt, followed by a reducing step with NaBH_4_ producing the formation of the heterogeneous hybrid. 

A full characterization of the hybrid was performed (Figure 1B–F). The wide-angle X-ray diffraction (XRD) confirmed the presence of Cu_2_O as mainly Cu species in this hybrid, by observation of four characteristic peaks, specifically at 37 degrees, corresponding to Cu_2_O, which matched well with JCPDS card no. 05-0667, and a minor fraction of Cu, matched with JCPDS card no. 04-0836 (Figure 1B). X-ray photoelectron spectroscopy (XPS) was used to characterize the surface composition and electronic states of these Cu catalysts, and the results confirmed the presence of Cu (Figure 1C and Figure S2). 

The high resolution XPS spectrum of Cu2p have peaks at 932.5 eV for Cu_2_O and peaks at 934.39 eV and 955.00 eV for CuO (Figure 1C and Figure S2), showed a certain oxidation in the surface of the nanoparticles. FT-IR analysis also confirmed the presence of Cu_2_O (Figure 1D). Transmission electron microscopy (TEM) analysis revealed mainly the formation of two size of spherical nanoparticles with a diameter average size 53 nm and 165 nm (Figure 1E,F and Figure S3). The inductively coupled plasma−optical emission spectroscopy results showed that the content of Cu in **G@TLL-Cu_2_O** was 6.4% (*w*/*w*).

Finally, the stability of the hybrid was evaluated at different temperatures, or in the presence of different additives, and no desorption of enzyme–metal system was observed in any case (Figure S4). 

### 3.2. Trichloroethylene (TCE) Degradation Catalysed by G@TLL-Cu_2_O Hybrid

Firstly, the possible adsorption of TCE to the G@TLL support was studied (Figure S5). In it, different concentrations of solvent: water were evaluated until there was no adsorption, the optimal point was 50:50 H_2_O:ACN (Figure S5A). In addition, other solvents were evaluated under the optimal conditions, showing that the optimal solvent was ACN, since there was still adsorption of TCE to the support (Figure S5B).

**Figure 2:**
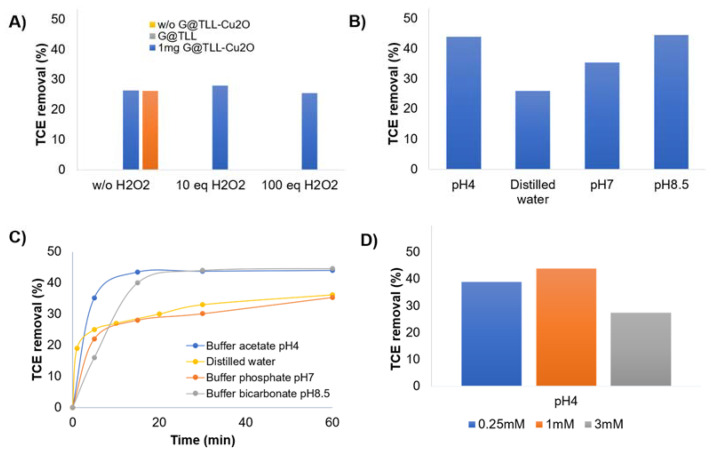
Degradation of 1mM of TCE in 60 min at r.t. and natural light. **(A**) Evaluation with different amount of G@TLL and **G@TLL-Cu_2_O** (1 or 10 mg) and in the presence of different amount of H_2_O_2_ (0–100eq); (**B**) Evaluation at different pH of the medium (50:50 aqueous medium: ACN) without H_2_O_2_; (**C**) Profile at different conditions without H_2_O_2_; (**D**) Evaluation of different amount of TCE initial at pH 4.

Thus, the reaction was carried out in aqueous media (50:50 H_2_O:ACN) at 25 °C. Initially, no conversion was obtained without catalyst or exclusively using G@TLL (Figure 2A). First, the degradation of TCE (1 mM) was studied with 1 mg of **G@TLL-Cu_2_O** hybrid (Figure 2A). TCE at 27% (35.5 mg/L) was degraded in 60 min without the production of toxic by-products (1,1-DCE and VC). To try to improve the degradation, the amount of catalyst was increased to 10 mg, although no changes were observed. Therefore, different amounts of a green oxidant such as H_2_O_2_ (33% *v*/*v*) were evaluated, and again, no significant differences were observed. This shows that the process is not a Fenton process.

**Scheme 1:**
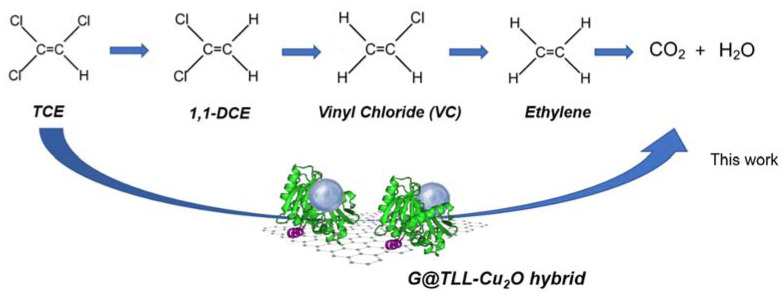
Mechanism of TCE degradation by **G@TLL-Cu_2_O** hybrid.

To evaluate the effects of pH on the catalytic efficiency of **G@TLL-Cu_2_O** in TCE degradation, four solutions with different pH values were studied, buffer sodium acetate pH 4, distilled water, buffer sodium phosphate pH 7, and buffer sodium bicarbonate pH 8.5 (Figure 2B). An increasing trend was observed as we increased the pH, obtaining a maximum degradation value of 44.5% (58.5 mg/L) at pH 8.5. However, at pH 4, a lower pH than distilled water, TCE degradation was similar to pH 8.5. Furthermore, the TCE degradation profile was analysed for these studies, demonstrating a clear rapid trend of TCE elimination in the first 5 min in the case of pH 4. At higher pHs the tendency in the first 5 min was slower (Figure 2C). At this point, the evaluation of the increase and decrease in the initial concentration of TCE was carried out (Figure 2D), where 3 mM and 0.25 mM of initial TCE were evaluated compared to the previous concentration already studied (1 mM), all at pH4. In this case, it was observed that by decreasing the amount of initial TCE, the conversion was similar, while when we increased the amount of TCE 3 times, the degradation decreased by half (Figure 2D). Therefore, the optimal amount of starting TCE was determined to be 1 mM. Therefore, the best conditions for direct degradation of TCE by the **G@TLL-Cu_2_O** hybrid were 1 mM of TCE, 1 mg catalyst, in 50:50 ACN: pH4 acetate buffer without H_2_O_2_ (Scheme 1).

**Figure 3:**
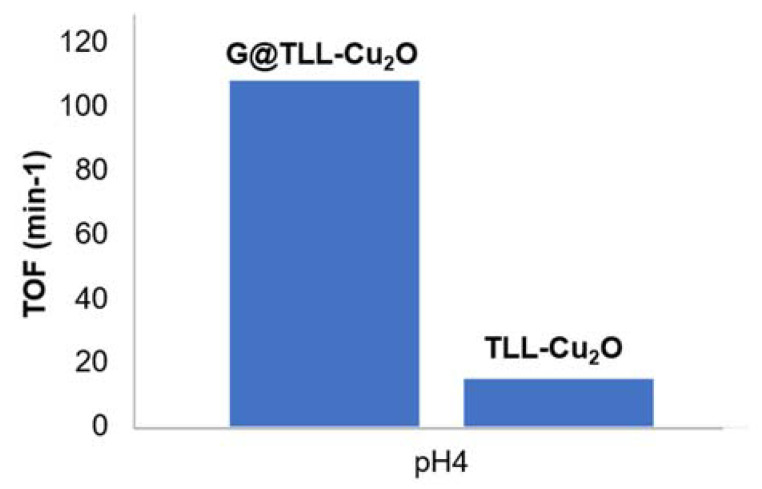
Comparative of TOF value (min^−1^) between **G@TLL-Cu_2_O** hybrid and TLL-Cu_2_O (catalyst no supported) at 50:50 ACN:buffer acetate pH4. This experiment was calculated at 5 min with 20–30% of TCE elimination.

Finally, a comparison, in TCE degradation at pH 4, was made between the **G@TLL-Cu_2_O** and non-supported TLL-Cu_2_O (see Supplementary Information) (Figure S6), to evaluate the advantage of the in situ formation of Cu_2_O nanoparticles on the immobilized derivative where the enzyme is fixed to graphene in open conformation, homogeneously distributed, and not in an aggregated form as exits in TLL-Cu_2_O hybrid. Figure 3 shows almost 8 times more efficiency for **G@TLL-Cu_2_O**, with a TOF value of 109 min^−1^ compared to 14.9 min^−1^ for TLL-Cu_2_O. 

### 3.3. Rhodamine B (RhB) Degradation Catalysed by G@TLL-Cu_2_O Hybrid

**Figure 4:**
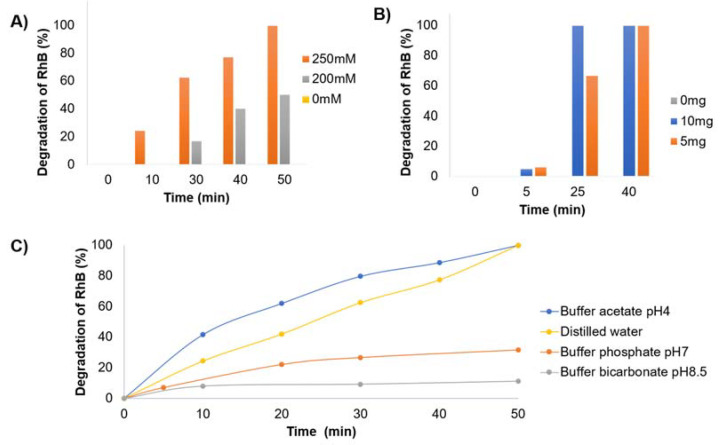
Degradation of 0.1 mM (48 ppm) of RhB at r.t. and natural light. (**A**) Effect of the amount of H_2_O_2_ with 5 mg of **G@TLL-Cu_2_O** hybrid; (**B**) Effect of the amount of **G@TLL-Cu_2_O** hybrid with 250 mM of H_2_O_2_; (**C**) Effect of medium pH, conditions: 50:50 ACN:Buffer with 5 mg of **G@TLL-Cu_2_O** hybrid and 250 mM of H_2_O_2_.

For the degradation of rhodamine B, firstly, the adsorption of RhB to G@TLL was also studied. The optimal conditions to avoid any unspecific adsorption of compound to graphene were 50:50 ACN:H_2_O (data not shown). The effects of several factors, such as the amount of hybrid, the amount of the green oxidant (H_2_O_2_) and the pH value of the reaction solution on the degradation of RhB were studied (Figure 4).

Initially, the amount of H_2_O_2_ was studied in a range from 0 mM to 250 mM (Figure 4A and Figure S7). Here, it is shown that at least 250 mM was necessary to complete degradation of RhB in 50 min. If we observe the result with 200 mM, a clear influence on the degradation is observed, since the drop in it is 50%. It was also tested with 500 mM H_2_O_2_, obtaining similar results to using 250 mM (data not shown). Then, the effect of the amount of **G@TLL-Cu_2_O** hybrid was evaluated at 250 mM of H_2_O_2_ (Figure 4B, Figures S7A and S8). In this case, complete degradation was achieved after 25 min incubation using 10 mg, with a 60% using 5 mg of catalyst. 

Finally, the ionic strength of the medium was evaluated by combining ACN and several buffers at different pH (50:50), these being acetate buffer pH 4, distilled water, phosphate buffer pH 7, and bicarbonate buffer pH 8.5 (Figure 4C, Figures S9 and S10). All of them were studied without catalyst, and no effect was observed (data not shown). The results showed a clear effect of pH in the degradation speed, where degradation is higher from basic to acidic media, being the best results at pH 4. A catalytic Fenton process of Cu is taking place, and OH· radicals in the medium are more active at lower pHs [33]. 

**Scheme 2:**
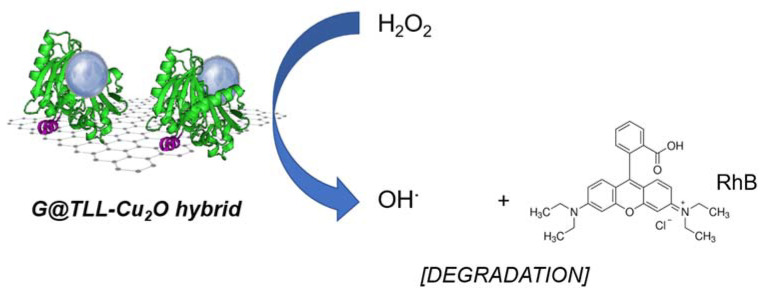
Schematic representation of RhB degradation in aqueous medium and formation of oxidative free radical by **G@TLL-Cu_2_O** hybrid.

Therefore, the best conditions obtained were 5 mg of catalyst, 250 mM H_2_O_2_ in an ACN medium: pH 4 acetate buffer (50:50) for a complete degradation of RhB (Scheme 2) in less toxic products, similarly to that recently reported [18].

To assess the advantage of this catalyst material, where synthesis and application were performed under very mild conditions, a comparison was made with other reported RhB catalytic degradation (Table 1). Most of the systems based on Cu_2_O nanoparticles published in the literature use high power ultraviolet light and visible light lamps. The first advantage was that the catalyst developed in this work was able to carry out RhB degradation in natural light. On the other hand, it was the only one capable of reaching 100% degradation in less than 1 h; those described in Table 1 needed more than 1 h or even 4 h with an initial amount of RhB up to 10 times less than the system developed in this work. Therefore, it can be seen how the best result corresponds to this work, since it was able to degrade 48 mg/L in 50 min under standard conditions in natural light.

## 4. Conclusions

Heterogeneous lipase-CuNPs hybrid catalysts were synthesized. *Thermomyces lanuginosus* lipase (TLL) was immobilized on a biographene preparation, and this immobilized preparation was used as a solid-phase scaffold for the in situ fabrication of Cu_2_O nanoparticles induced by lipases molecules, thus creating a successful G@TLL-Cu_2_O hybrid. The formation of Cu_2_ONPs was demonstrated by XRD and XPS where it was seen that it was the majority species. The TEM microscopy showed that the Cu_2_ONPs were homogeneously distributed over the G@TLL surface with sizes of 53 nm and 165 nm. 

**G@TLL-Cu_2_O** hybrid was successfully used in the direct degradation of trichlorethylene (TCE), a toxic organic chloride. An amount of 60 ppm of TCE was degraded in 60 min at pH 4 in aqueous solution and room temperature without formation of other toxic by-products. In addition, a TOF value of 7.5 times higher than the unsupported counterpart (TLL-Cu_2_O) was obtained, demonstrating the improvement of the catalytic efficiency of the solid-phase system. In addition, another toxic organic compound was evaluated, Rhodamine B (RhB). The hybrid presented an excellent catalytic performance for the degradation of RhB obtaining complete degradation (48 ppm) in 50 min in aqueous solution, at room temperature, and with the presence of a green oxidant such as H_2_O_2_ by a Fenton process. 

These excellent results in the degradation processes of toxic organic compounds open up potential applications in the field of energy and the environment, specifically, in water treatment systems, in water remediation which avoids the exposure of said pollutants to human health, and in addressing the global ecological problem.

## Figures and Tables

**Table 1:** Comparative in the degradation of Rhodamine B (RhB) by different authors.

Catalyst	Cu (%*w*/*w*)	Method	[RhB] (mM)	[H_2_O_2_] (mM)	Catalyst (g/L)	Time (min)	RhB Removal (%)	Ref
**G@TLL-Cu_2_O hybrid**	6.4	Natural light	0.1	250	2.5	50	100	This work
**Cu_2_O@3D-rGO@NCS nanocomposite**	nd	AM 1.5G filter, 500 W Xe lamp	0.01	-	0.2	150	90	[23]
**SiNWAs/Cu_2_O heterojunctions**	65.11	Xe lamp irradiation with a cut-off filter (l > 420 nm)	0.02	160	nd	60	100	[34]
**Cu_2_O/RGO-3**	nd	500-W high-pressure Hg arc lamp	0.01	-	0.4	120	90	[35]
**Cu_2_O NPs**	nd	UV light (250 W)	0.01	-	20	220	100	[18]
**CuONPs**	nd	Fluorescent lamp	0.01	-	1	150	83	[24]
**Ni-Cu@MWCNTs**	nd	UV light	0.04	0.12 + [Fe^2+^]	0.2	50	98	[36]

## Data Availability

The authors confirm that the data supporting the findings of this study are available within the article and/or its Supplementary Materials.

## Supplementary Materials

Supplementary material related to this article can be found, in the online version: https://www.mdpi.com/article/10.3390/nano13030449/s1. Additional experimental details. Figure S1: Three-dimensional Surface of dimer from TLL and immobilization curve of G@TLL. Figures S2 and S3: additional images of characterization (XPS, XRD, TEM) of G@TLL-Cu2O hybrid. Figure S4: analysis of stability of G@TLL-Cu2O hybrid. Figure S5: Study of adsorption of TCE to G@TLL. Figure S6: Characterization of TLL-Cu2O hybrid Figures S7–S10: Additional experiments in degradation of RhB catalyzed by G@TLL-Cu2O hybrid.
