# Supplementary material for: Graphene-TLL-Cu2ONPs Hybrid as Highly Efficient Catalyst for Degradation of Organic Compounds
Source: Nanomaterials (Basel). 2023 Jan 21;13(3):449. doi: 10.3390/nano13030449 (PMC9921335; doi:10.3390/nano13030449)
Supplement: Supplementary file 1 [file nanomaterials-13-00449-s001.zip › nanomaterials-2158531-supplementary.pdf]

Supplementary information

Graphene-TLL-Cu_2ONPs Hybrid as Highly Efficient Catalyst for Degradation of Organic Compounds

Noelia Losada-Garcia, Jannier Carranza and Jose M. Palomo *

Instituto de Catálisis y Petroleoquímica (ICP), CSIC, Marie Curie 2, 28049 Madrid, Spain;
* Correspondence: josempalomo@icp.csic.es

Synthesis of TLL-Cu_2O hybrid

0.9 mL of commercial (18 mg of protein) *Thermomyces lanuginosus* lipase (TLL) solution was added to 60 mL 0.1M of buffer sodium phosphate pH 7 in a 250 mL glass bottle containing a small magnetic bar stirrer. Then, 600 mg of Cu_2SO_4 x $5H_2O$ (10 mg/ml) was added to the protein solution and it was maintained for 16 hours. After the first 30 min incubation, the solution turned cloudy (turquoise). After 16 h, 6 mL of $NaBH_4$ (300 mg) aqueous solution (1.2 M) was added to the cloudy solution (in two times of 3 mL) obtaining a final concentration of 0.12 M of sodium borohydride in the mixture. The solution turned rapidly black and, the mixture was reduced during 30 min. After the incubation, the mixture was centrifuged at 8000 rpm for 5 min, (10 mL per falcon type tube). The generated pellet was re-suspended in 15 mL of water. It was centrifuged again at 8000 r.p.m for 5 min and the supernatant removed. The process was repeated twice more. Finally, the supernatant was removed and the pellet of each falcon was re-suspended in 2 mL of water, collected all solutions in a round-bottom flask, frozen with liquid nitrogen and lyophilized for 16 hours. After that, 150 mg of the so called **TLL-Cu_2O hybrid** was obtained.

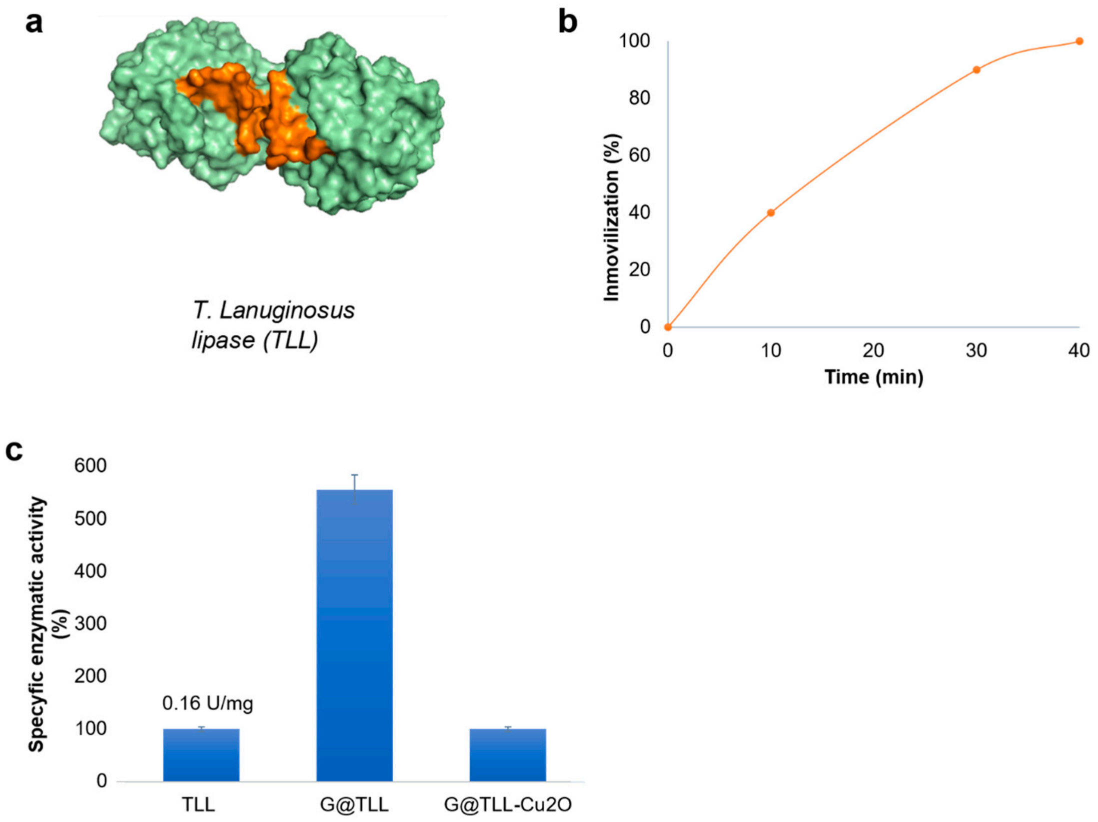

Figure S1. A) Three-dimensional Surface of dimer from TLL; **B)** Immobilization curve of G@TLL; **C)** Enzymatic activity.

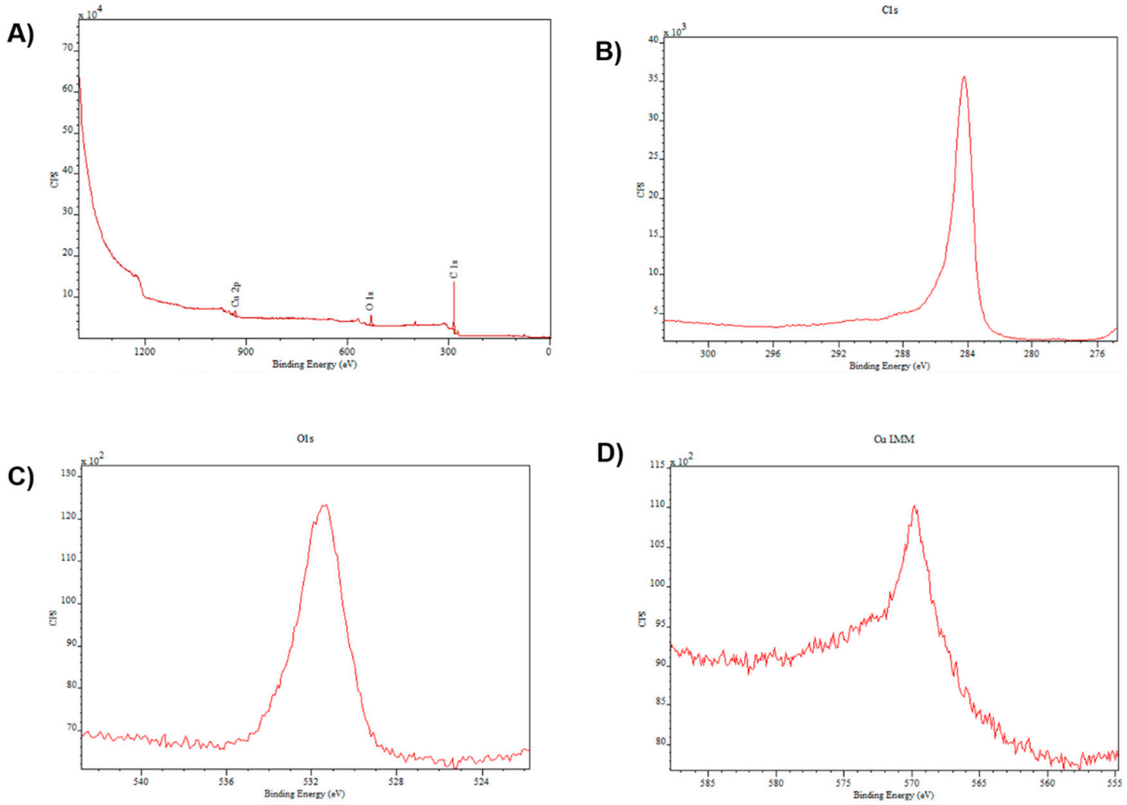

Figure S2. XPS analysis of **G@TLL-Cu_2O** hybrid. **A**) XPS full spectrum. **B)** XPS spectrum of C1s, **C**) XPS spectrum of O1s. **D**) Cu LMM auger spectrum.

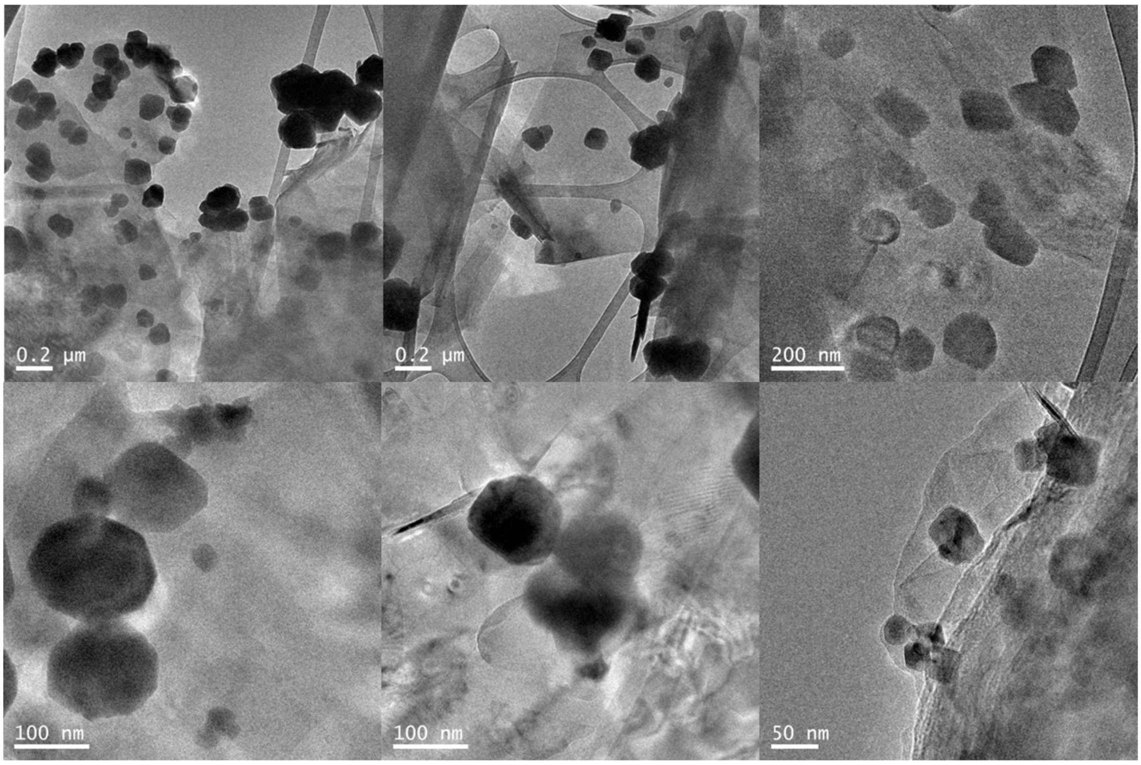

Figure S3. TEM images of **G@TLL-Cu_2O** hybrid.

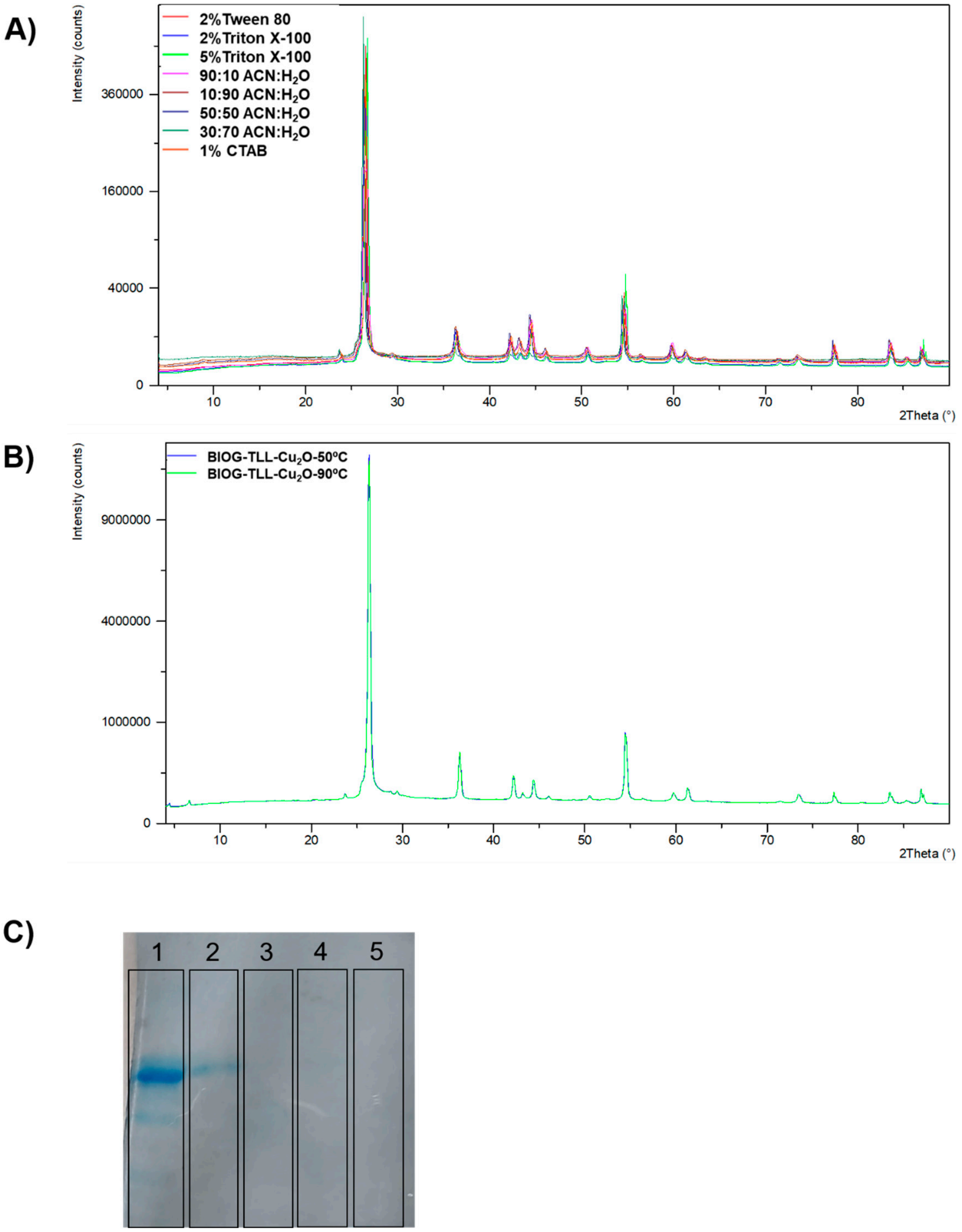

Figure S4. A) XRD spectra of the **G@TLL-Cu_2O** hybrid incubated in different solvents and detergents; **B)** XRD spectra for **G@TLL-Cu_2O** hybrid in SDS at 50 and 90 ºC; **C) 1**-TLL, **2-** G@TLL, **3- G@TLL-Cu_2O** pre-treated with SDS at 90ºC, **4- G@TLL-Cu_2O** pre-treated with SDS at 50ºC, **5- G@TLL-Cu_2O**.

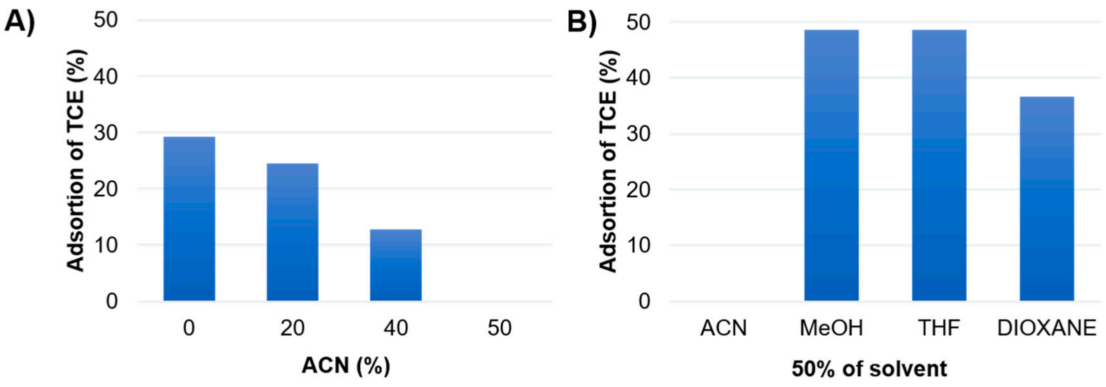

Figure S5. Study of adsorption of TCE to G@TLL in 1min. **A)** Different concentrations of ACN; **B)** Different solvents at 50:50 with water.

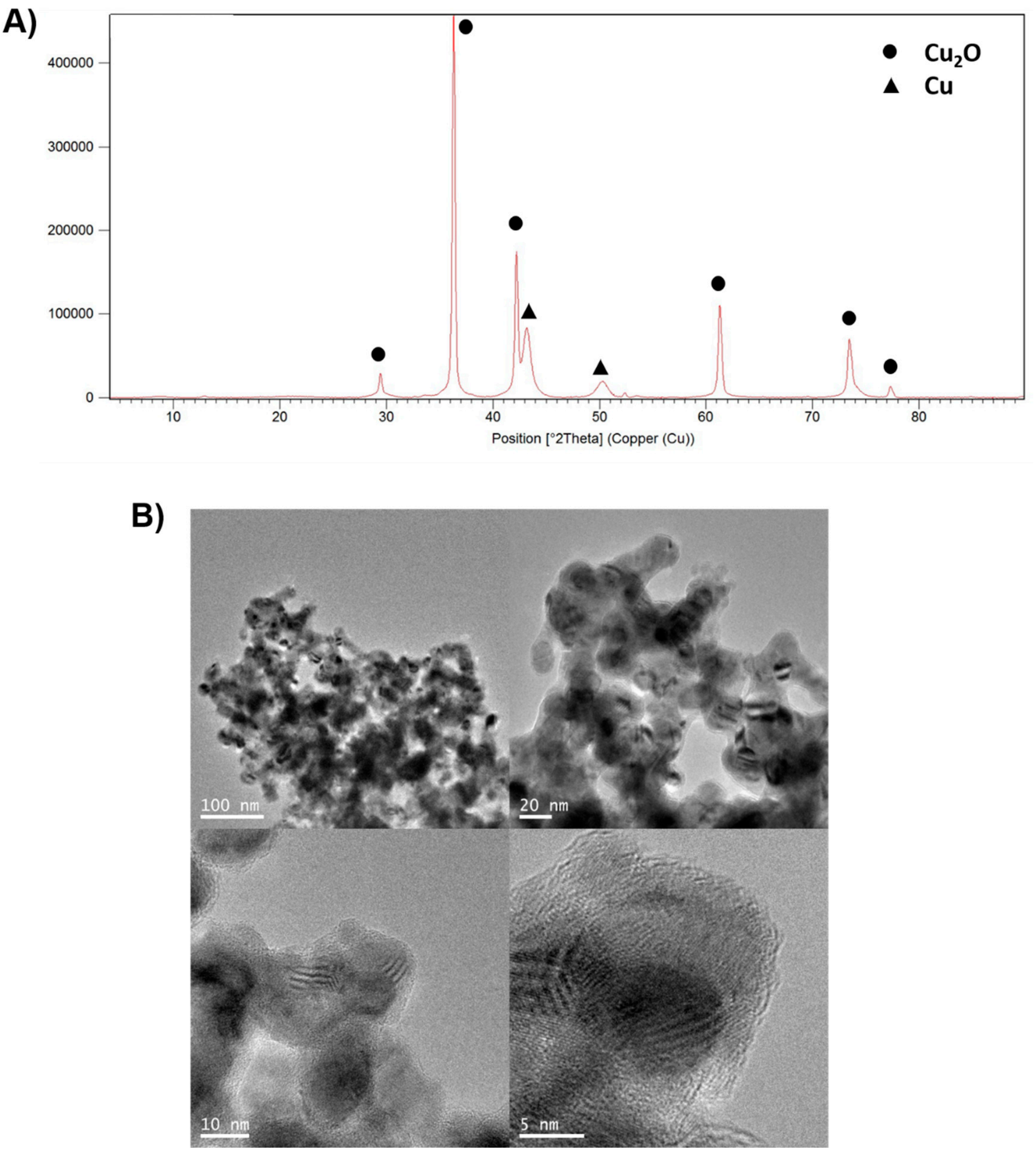

Figure S6. Characterization of **TLL-Cu_2O** hybrid: **A)** XRD spectrum; **B)** TEM and HR-TEM images.

A)

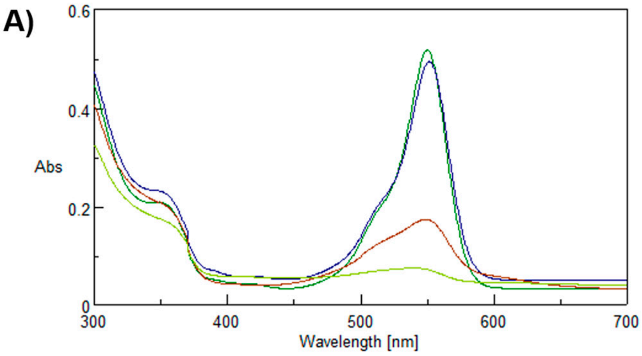

Time (min)	Absorbance	Degradation (%)
0	0.51	0
10	0.49	4.5
30	0.18	66.4
40	0.005	>99

B)

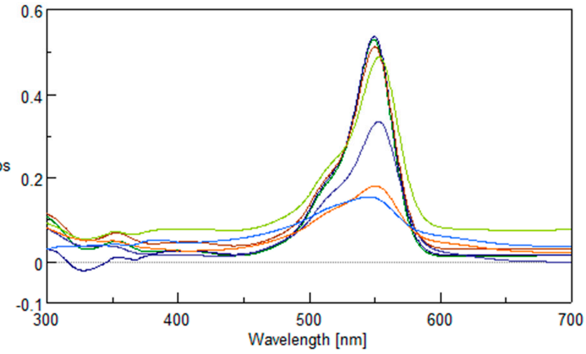

Time (min)	Absorbance	Degradation (%)
0	0.53	0
10	0.53	0
20	0.52	1.5
30	0.48	8
40	0.33	38
50	0.2	62
60	0.15	71

Figure S7. Effect of the amount of H_2O_2 with 5mg of **G@TLL-Cu_2O** hybrid and 0.1mM of RhB: **A)** 250mM; **B)** 200mM (inset table of data).

A)

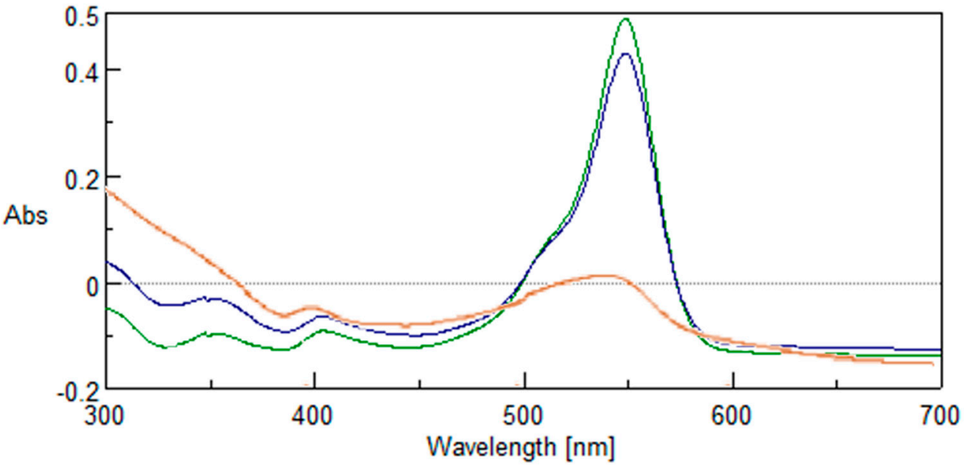

B)

Time (min)	Absorbance	Degradation (%)
0	0.47	0
5	0.42	10
25	0.008	99

Figure S8. A) Spectra of degradation of 10mg of **G@TLL-Cu_2O** hybrid with 250mM of H_2O_2 and 0.1mM of RhB; **B)** Table of data.

A)

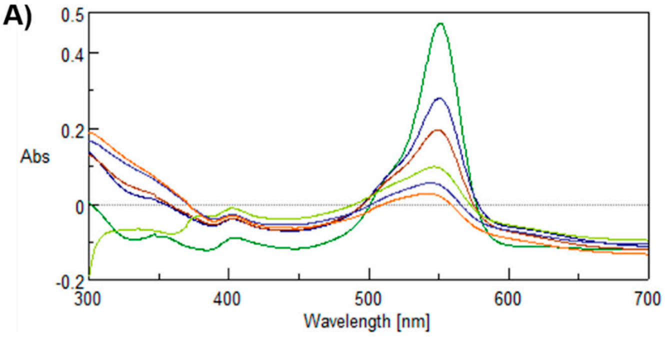

Time (min)	Absorbance	Degradation (%)
0	0.48	0
10	0.28	41
20	0.2	59
30	0.1	80
40	0.05	88
50	0.01	>99

B)

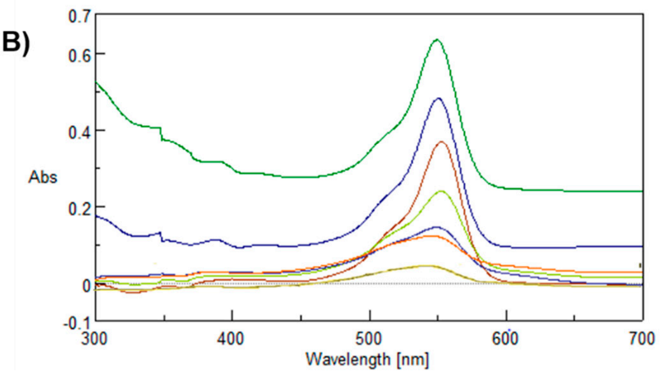

Time (min)	Absorbance	Degradation (%)
0	0.63	0
10	0.48	24
20	0.37	42
30	0.23	63
40	0.14	78
45	0.09	85
50	0.02	99

Figure S9. Effect of medium pH in the degradation of 0.1mM of RhB, conditions: 50:50 ACN:Buffer with 5mg of **G@TLL-Cu_2O** hybrid and 250mM of H_2O_2: **A)** Buffer sodium acetate pH4; **B)** Distilled water (inset table of data).

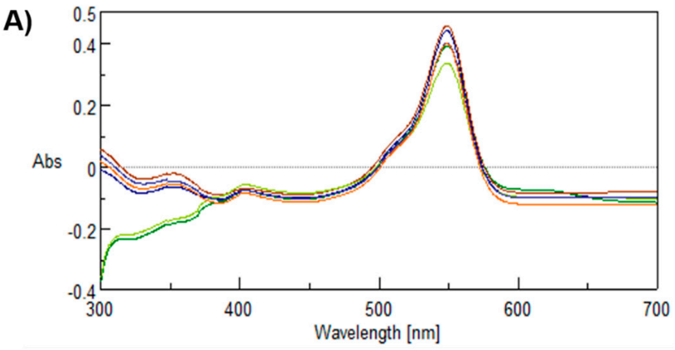

Time (min)	Absorbance	Degradation (%)
0	0.45	0
5	0.43	4
20	0.35	22
30	0.33	26
50	0.3	30

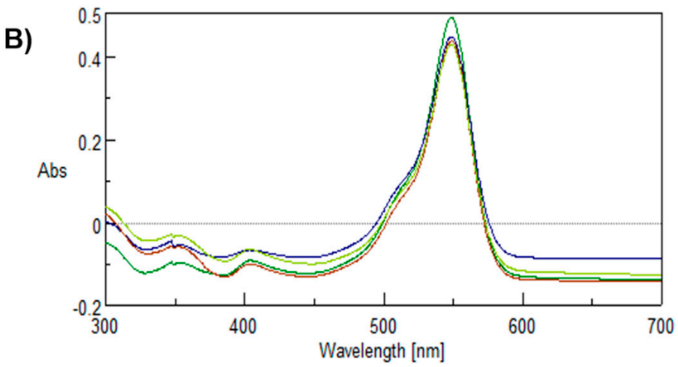

Time (min)	Absorbance	Degradation (%)
0	0.48	0
10	0.44	9.3
30	0.43	11.2
50	0.42	13.4

Figure S10. Effect of medium pH in the degradation of 0.1mM of RhB, conditions: 50:50 ACN:Buffer with 5mg of **G@TLL-Cu_2O** hybrid and 250mM of H_2O_2: **A)** Buffer sodium phosphate pH7; **B)** Buffer sodium bicarbonate pH8.5.
